# Acute myeloid leukemia-derived extracellular vesicles induced DNA methylation changes responsible for inflammatory program in normal hematopoietic stem progenitor cells

**DOI:** 10.3389/fimmu.2025.1569159

**Published:** 2025-04-10

**Authors:** Daniela Lamorte, Giovanni Calice, Stefania Trino, Michele Santodirocco, Antonella Caivano, Luciana De Luca, Ilaria Laurenzana

**Affiliations:** ^1^ Laboratory of Preclinical and Translational Research, IRCCS Centro di Riferimento Oncologico della Basilicata (CROB), Rionero in Vulture (PZ), Italy; ^2^ Trasfusional Medicine Department, Puglia Cord Blood Bank (CBB), Fondazione IRCCS Casa Sollievo della Sofferenza Hospital, San Giovanni Rotondo (FG), Italy; ^3^ Unit of Clinical Pathology, IRCCS Centro di Riferimento Oncologico della Basilicata CROB, Rionero in Vulture (PZ), Italy

**Keywords:** acute myeloid leukemia, extracellular vesicles, hematopoietic stem and progenitor cells, DNA methylation, differentially expressed genes, inflammation, hematopoiesis, leukemogenesis

## Abstract

**Introduction:**

Acute Myeloid Leukemia (AML) cells communicate with surrounding normal cells, including hematopoietic stem progenitor cells (HSPCs), in the bone marrow, and modify their fate supporting tumor growth. This communication can be mediated by Extracellular Vesicles (EVs), small vectors carrying a range of tumor molecular information. One of the hallmarks of AML is the aberrant DNA methylation. It is not known if and how AML cells can modify the epigenomic profile of healthy HSPCs. Here, we investigated the DNA methylation profile of HSPCs after exposure to AML derived-EVs.

**Methods:**

Cord blood derived-HSPCs were treated with AML cell line derived-EVs for 20 hours and then their DNA methylation profile was analyzed by methylation array. We cross-referenced differential methylated genes (dmGs) with differential expressed genes (deGs) obtained by gene expression profile of same EV treated-HSPCs. Gene ontology was performed on dmGs and deGs. To confirm the expression of some genes, digital PCR was applied.

**Results:**

AML-EVs induced DNA methylation changes in HSPCs after short time exposure, showing 110-890 dmGs. In particular, we reported a DNA hypo-methylation in both promoter and body regions. DmGs showed an enrichment in hematopoietic and immunological processes, inflammation, cell movement and AML pathways. The intersection between dmGs and deGs identified 20 common genes, including DSE, SEMA4A, NFKB1 and MTSS1, whose over-expression could be associated with the hypo-methylation of their gene body, and other ones, such as SLA and CUTA whose down-expression could be associated with the hypo-methylated promoter. These deGs were involved in NF-kB pathway, interleukin mediate Toll like receptor signaling and, of note, in tumor.

**Conclusion:**

This study is the first proof-of-concept that AML-EVs were able to induce changes in DNA methylation of HSPCs modulating the expression of genes involved in inflammatory processes capable of modifying normal hematopoiesis towards leukemic like processes.

## 1 Introduction

Acute myeloid leukemia (AML) is a hematological neoplasm characterized by uncontrolled proliferation of myeloid blasts evolved from hematopoietic stem progenitor cells (HSPCs) in the bone marrow (BM) and peripheral blood. Past discoveries have contributed to the understanding of genetic defects and biological changes in myeloid cells that underlie disease onset and progression. Recent advances in genomic sequencing allowed the identification of several new recurrent genetic mutations, leading to improvements in prognosis and molecular characterization within AML different subgroups (1). It is now known that, in addition to genetic mutations, also epigenetic modifications, including DNA methylation, are involved in AML pathogenesis.

DNA methylation regulates gene expression potential through mechanisms that do not alter the genome sequence leading to leukemogenesis through silencing of tumor suppressors and activating oncogenes (2, 3). Generally, DNA hyper-methylation in gene promoter mediates its silencing, while hypo-methylation is associated with activated gene expression (4). The extensive analysis of gene-wide DNA methylation in hematopoietic lineage cells showed that DNA methylation is essential in hematopoiesis and its dysregulation is a critical event in the initiation and progression of AML (5, 6). DNA methylation is catalyzed by DNA methyltransferases (DNMTs) while DNA demethylation relies on ten-eleven translocation proteins (TETs) (7, 8) and can be highly cell-type specific and dynamic (9).

Extracellular Vesicles (EVs) are subcellular plasma membrane-enclosed particles measuring 0.02–2 µm and which are released, through a complex budding mechanism, by all type of cells *in vitro* and *in vivo*. Their cargo, which includes bioactive lipids, nucleic acids, proteins and signaling nucleotides, reflects the state of original cells and can be transfer to proximal and distal recipient cells by influencing their physiological behavior (10–13). It was recently demonstrated that EVs, delivering epigenetic regulators including microRNAs, DNMTs and TETs (14, 15), can reprogram the epigenomic profile by remodeling DNA, RNA and histones in recipient cells (16, 17). For example, EVs of acute coronary syndrome patients modulated gene expression through *de novo* DNA methylation signals in peripheral blood mononuclear cells (18). Moreover, DNMT1 mRNA transcript by leukemic EVs may reprogram normal hematopoietic cells (14). All these findings have opened a new era in the field of intercellular communication and resultant phenotype changes observed in tumors, cell biology, diagnosis and therapeutics.

In AML, it is well known that leukemic cells interact and influence, through EVs, the fate of neighboring cells including HSPCs, immune cells and other ones in BM (19, 20).

In this context, we recently demonstrated that AML-EVs, releasing microRNAs, were able to inhibit HSPC differentiation, to redistribute HSPC populations and to activate an HSPC inflammatory program (21).

In this paper, we reported, for the first time, the analysis of DNA methylation of HSPCs after their *in vitro* exposure to AML-EVs demonstrating that these ones modified HSPC methylome.

## 2 Materials and methods

### 2.1 AML-EVs and HSPCs

AML-EVs were isolated from KG-1 AML cell line and characterized as previously reported (21). As normal HSPCs, primary CD34^+^ cells were purified from three pools of umbilical cord blood (UCB) bags as previously reported (21) and three independent experiments were conducted as group a, group b and group c. Specifically, CD34^+^ of group a derived from a pool of 10 UCB bags collected in 3 days; CD34^+^ of group b derived from a pool of 8 UCB bags collected in 5 days, and CD34^+^ of group c derived from a pool of 15 UCB bags collected in 3 days.

Briefly, HSPCs were treated with AML-EVs or with filtered phosphate buffered saline (PBS) as control (Ctrl) for 20 hours as previously reported (21). After treatment, HSPCs were harvested to analyze the HSPC progenitor distribution by flow cytometer. In particular, HSPCs included hematopoietic stem cells (HSC), multipotent progenitors (MPP), lymphoid-primed multipotent progenitors (LMPP), multi-lymphoid progenitors (MLP), common myeloid progenitors (CMP), granulocyte-macrophage progenitors (GMP), megakaryocyte-erythroid progenitors (MEP) (**Table 1**).

**Table 1:** Progenitor distribution of control HSPCs in three control groups post 20 hours.

Ctrl	HSC	MPP	LMPP	MLP	CMP	MEP	GMP
**a**	3.2%	2.4%	1.3%	1.17%	58.7%	4.9%	26.7%
**b**	8.6%	7.8%	1.55%	0.9%	55.8%	7.17%	15.3%
**c**	5.3%	3.5%	0.7%	0.2%	61.2%	9.7%	16.6%

### 2.2 HSPC-DNA isolation

DNA was extracted from HSPCs, treated or not with AML-EVs, by AllPrep DNA/RNA Micro Kit (Qiagen GmbH, Hilden, Germany) following manufacturer’s instructions. DNA quality was controlled by agarose gel electrophoresis and quantified by a NanoDrop ND-1000 Spectrometer (Thermo Scientific, Wilmington, DE, USA).

### 2.3 Bisulfite conversion and array−based DNA methylation

Genomic DNA (300 ng) was treated with sodium bisulfite using the Zymo EZ DNA Methylation Kit (Zymo Research, Orange, CA, USA) according to the manufacturer’s instructions, with the alternative incubation conditions recommended for the Illumina Infinium Methylation Assay (Illumina, CA, USA). Array-based DNA methylation was performed according to the Infinium HD Methylation Assay protocol and Infinium Human Methylation EPIC (850 k) BeadChip (Illumina), as previously described (22). Bead-Chips were scanned using the Illumina HiScanSQ system (Illumina).

### 2.4 Microarray data and functional enrichment analysis

Illumina Infinium Human Methylation EPIC 850K raw data were imported to R/Bioconductor environment for processing according to Todoerti et al. (23). Quality control, filtering by detection *p*-value, SWAN normalization and other filters (SNPs probes/loci, X or Y chromosomes localized probes/loci, Cross-Reactive probes/loci were applied (24, 25).

Subsequently, Beta- and M-values were computed. Beta-value, as ratio of fluorescence intensity of methylated vs all probes, was used for visualization and plotting; M-value, as log2 ratio of methylated vs unmethylated probe signals, was used for differential methylation analysis of EV-treated Group vs Ctrl Group (the significant cut-off was established on false discovery rate, FDR, *q*-value < 0.05). No differences with FDR < 0.05 were found between EV treated-cells Group vs Ctrl Group.

Principal Component Analysis (PCA) was plotted to visualize pattern of relationship among all samples and performed on the M-value methylation level; the analysis points up the relations based on the probes with sd/mean ratio > 0.5 (probes size = 32089).

Our new approach was an analysis based on computation of the non-parametric coefficient of variation of Beta-value for each sample on the respective control. The resulting lists were filtered on the 3rd Quartile, so only probes be part of the upper 25% were considered for next analyses.

Thus, we obtained the most variable probes for each group (a, b and c), treated vs Ctrl, which were further filtered on the absolute delta Beta-value > 0.2, obtaining differentially methylated CpGs (dmCpGs).

The UCSC RefGene Name, UCSC RefGene Group, Islands Name and Relation to Island annotations were referred to the dmCpCgs. The Promoter region included TSS200, TSS1500 and 5’UTR, while Body region included 1st exon, gene body, 3’UTR and exon boundaries.

Afterward, to select common genes within a, b and c groups, hypo- and hyper-methylated genes were intersected and were highlighted in Venn diagrams. Subsequently, as reported in our previous work (21), we proceeded to the enrichment analysis of the Genes’ lists, by clusterProfiler package (Bioconductor – clusterProfiler) among MSigDB gene sets collections selecting “C2: curated gene sets” which include “CGP: chemical and genetic perturbations” and “CP: Canonical pathways” (GSEA | MSigDB | Human MSigDB Collections); the significant cut-off of the enrichment results is the adjusted *p*-value < 0.05. Moreover, common genes, resulting from intersection of three or two groups, were combined with genes resulted by GEO GEP Dataset submitted, GSE189492, mentioned above (21). The GEP data previously reported (GSE 189492, doi: 10.3389/fonc.2022.824562) and used in this paper were obtained by the same cells (CTRL_a, _b, _c and EVs_a, EVs_b, EVs_c) on which we have performed DNA methylation analysis.

All analyses were implemented by R/Bioconductor software and CRAN packages (https://www.r-project.org/; https://www.bioconductor.org/; https://cran.r-project.org/).

The resulted 20 genes, were evaluated by ShinyGO 0.77 [ShinyGO 0.77 182 (sdstate.edu)], selecting different pathway databases including KEGG, Panther, Reactome and WikiPathways, with 0.05 FDR cut-off and 10-2000 pathway size.

### 2.5 RNA Extraction from HSPCs, reverse transcription and droplet digital PCR

Total RNA was extracted from HSPCs, treated or not with AML-EVs, by RNA/DNA/PROTEIN Purification Plus Micro Kit (Norgen Biotek Corporation, Canada) and quantified using the Nanodrop Spectrophotometer (Thermo Scientific, Wilmington, DE, USA) as previously reported (21). Reverse transcription of 500 ng RNA was performed using High-Capacity cDNA Reverse Transcription Kit (Applied Biosystems) as previously reported (21). The expression levels of HSPC-mRNAs (CUTA, SLA, DSE, SEMA4A, NFKB1, MTSS1) were evaluated by QX200 ddPCR System (Bio-Rad Laboratories, Hercules, CA). Hs00360405_g1 (CUTA), Hs00277129_m1 (SLA), Hs01897959_s1 (DSE), Hs00223617_m1 (SEMA4A), Hs00765730_m1 (NFKb), Hs00207341_m1 (MTSS1) Taq man assay (Thermo Fischer Scientific) were used in ddPCR.

## 3 Results

### 3.1 AML-EV exposure induced a modification of HPSC methylome

To understand whether leukemia EVs could induce methylome changes in normal HSPCs, we analyzed their DNA methylation pattern after *in vitro* AML-EV exposure in three independent experiments (here referred as group a, b and c).

**Figure 1:**
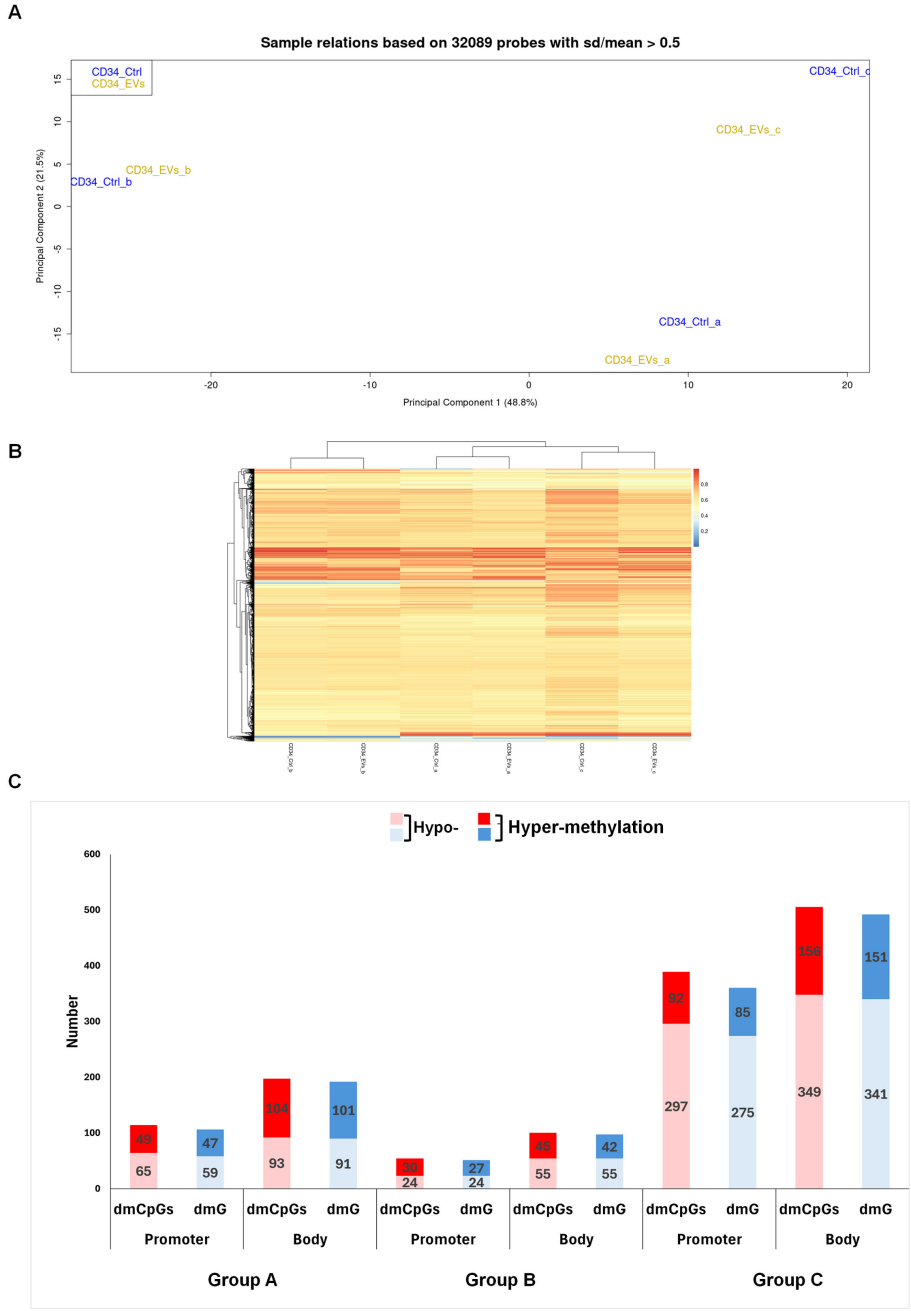
Methylation profile of HSPCs after AML EVs exposition. **(A)** Principal Component Analysis of 32089 probes of not treated (CD34_Ctrl) and AML EVs treated (CD34_EVs) HSPCs. Samples are annotated by experiments a, b and c. **(B)** Heat map of DNA methylation resulting from an unsupervised cluster analysis of 32089 probes. The probe clustering is reported on the left of the heatmap. The methylation level is expressed as a β-value ranging from 0 (blue) to 1 (red). **(C)** Column plot of Promoter and Body regions distinct in hypo- and hyper-methylated differential methylated CpGs (dmCpGs) (light and dark red) and differential methylated Genes (dmGs) (light and dark blue, respectively) of Group a, b and c.

PCA performed on the 32089 most variable probes of all HSPCs treated with AML-EVs and respective controls showed their separation into two clusters on principal component 1 (x-axis). Specifically, the first cluster consisted of CD34_ctrl and CD34_EVs of group b, the second one of CD34_ctrl and CD34_EVs of group a and c (**Figure 1A**). Moreover, each EV-treated HSPCs (CD34_EVs_a or CD34_EVs_ b or CD34_EVs_c) grouped with their respective control (CD34_ctrl_a or CD34_ctrl_ b or CD34_ctrl_c) (**Figure 1A**). The same results were observed in unsupervised cluster analysis of same probes (**Figure 1B**).

Therefore, we compared DNA methylation pattern of each EV-treated HSPCs with its matched control, identifying 311, 154 and 894 differentially methylated CpGs (dmCpGs) in group a, b and c, respectively with a prevalence of the hypo-methylated dmCpGs respect to hyper-methylated ones in both promoter and body regions, with a greater presence in the body in all groups (i.e. a: 197 vs 114; b: 100 vs 54; c: 505 vs 389). DmCpGs were distributed in the genome regions with this level of methylation: 114 dmCpGs in the group a (65 hypo- and 49 hyper-methylated), 54 dmCpGs in the group b (24 hypo- and 30 hyper-methylated) and 389 dmCpGs in the group c (297 hypo- and 92 hyper-methylated) were observed in the promoter. Instead, in the body region, 197 dmCpGs were found in group a (93 hypo- and 104 hyper-methylated), 100 dmCpGs in group b (55 hypo- and 45 hyper-methylated) and 505 dmCpGs in group c (349 hypo- and 156 hyper-methylated) (**Figure 1C**).

Detailing all groups, according to the genomic annotation, dmCpGs were distributed in island, shelves, shores and openSea (**Supplementary Figure 1A**, **2A**, **3A**); the most abundant dmCpGs were in open sea. Moreover, the annotation of dmCpGs in different genomic regions such as 5’UTR, TSS1500, TSS200, 1^st^ exon, 3’UTR, Body and Exon Boundaries showed higher hypo-methylation in group c (**Supplementary Figure 3B**). Detailing, a considerably higher hypo-methylation of CpGs can only be observed in group c (72% vs. 28%; **Supplementary Figure 3A**), while in groups a and b, hypo- and hyper-methylation are quite balanced (51% vs. 49%; **Supplementary Figures 1A**, **2A**). Moreover, among the different genomic regions examined, several regions exhibit higher degrees of hyper-methylation (**Supplementary Figures 1**-**3B**).

Afterward, we converted both promoter and body region dmCpGs of three groups a, b and c in their respective differentially methylated genes (dmGs) (**Figure 1B**). We observed a direct correspondence between hypo- and hyper- dmCpGs and hypo- and hyper-dmGs in all three groups.

### 3.2 DmGs in AML-treated HSPCs were involved in tumor inflammatory program

**Figure 2:**
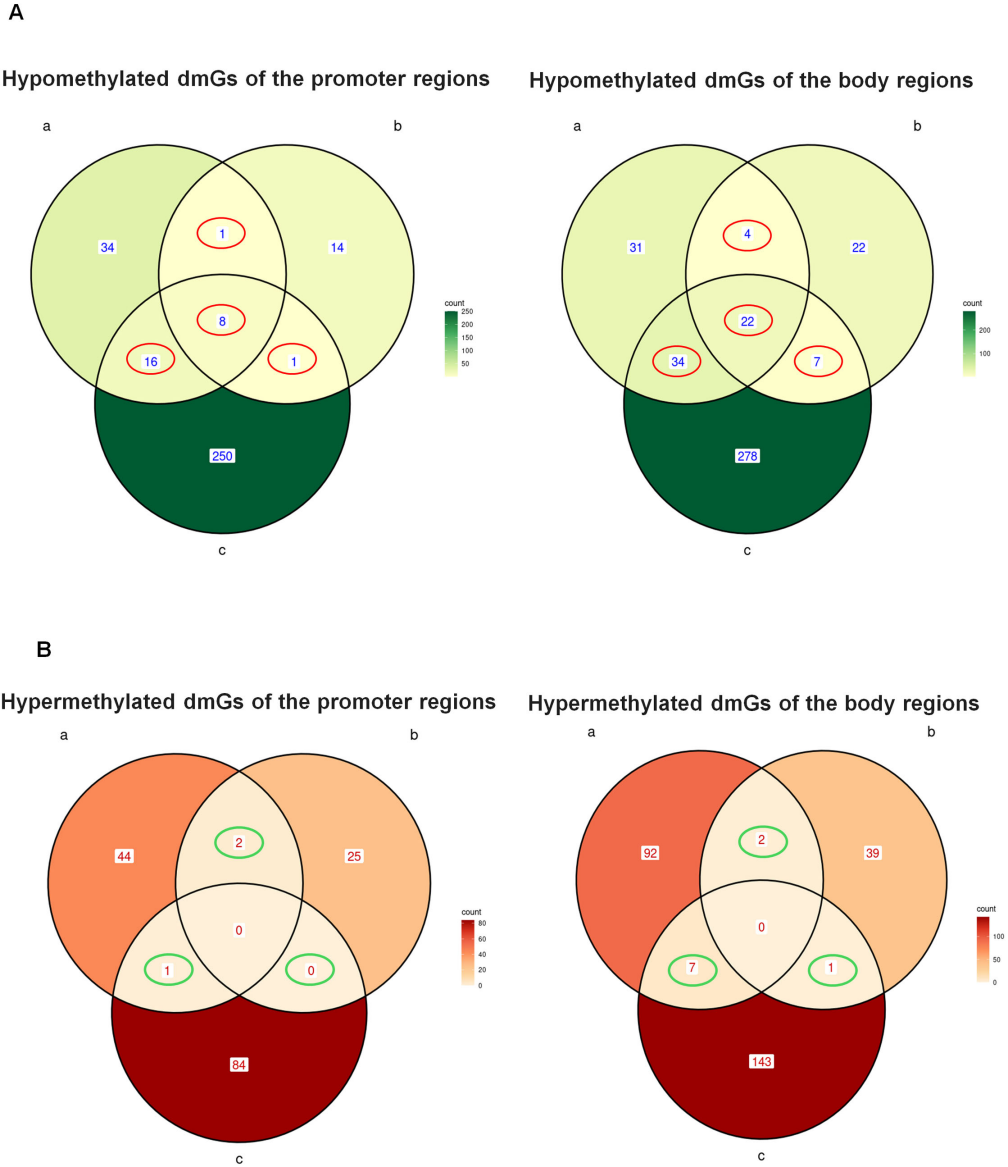
Common hypo- and hyper-methylated dmGs in promoter and body regions. Venn diagram shows the number of hypo- **(A)** and hyper-methylated **(B)** dmGs common to both promoter and body regions of the three groups a, b and c The red circles in **(A)** indicate the common hypo-methylated dmGs belonging to 2 (Promoter: n.1 a and b, n.1 b and c, n.16 a and c; Body: n.4 a and b, n.7 b and c, n.34 a and c) and 3 (Promoter n.8 a and band c, Body n.22 a and b and c) group intersections. The green circles in **(B)** indicate the common hyper-methylated dmGs belonging to 2 (Promoter: n.2 a and b, n.0 b and c, n.16 a and c; Body: n.2 a and b, n.1 b and c, n.7 a and c) or 3 (Promoter: n.0 a and b and c, Body n.0 a and b and c) group intersections.

To obtain a gene signature of AML-EV treated HSPCs, we intersected hypo- and hyper- dmGs from both promoter and body regions of the three groups to identify the common ones (**Figure 2**). We found 106 common dmGs across the three groups: 93 hypo-dmGs and 13 hyper-dmGs. In particular, for hypo-dmGs, in promoter regions we found 8 hypo-dmGs common to all groups, 16 hypo-dmGs common among group a/c and only one common in the comparison of groups a/b and b/c, while in the body region, 22 hypo-dmGs common genes were found among three groups, 4 genes among groups a/b, 34 genes among groups a/c and 7 among b/c (**Figure 2A**, **Supplementary Tables 1**, **2**). For hyper-dmGs, in promoter regions no hyper-dmGs were found between the three groups, while 2 genes were reported as common among group a/b and only one among group a/c while in the body region no common hyper-dmGs were found between the three groups, while 2 common genes were found among groups a/b, 7 among a/c and only one among b/c (**Figure 2B**, **Supplementary Tables 3**, **4**).

**Figure 3:**
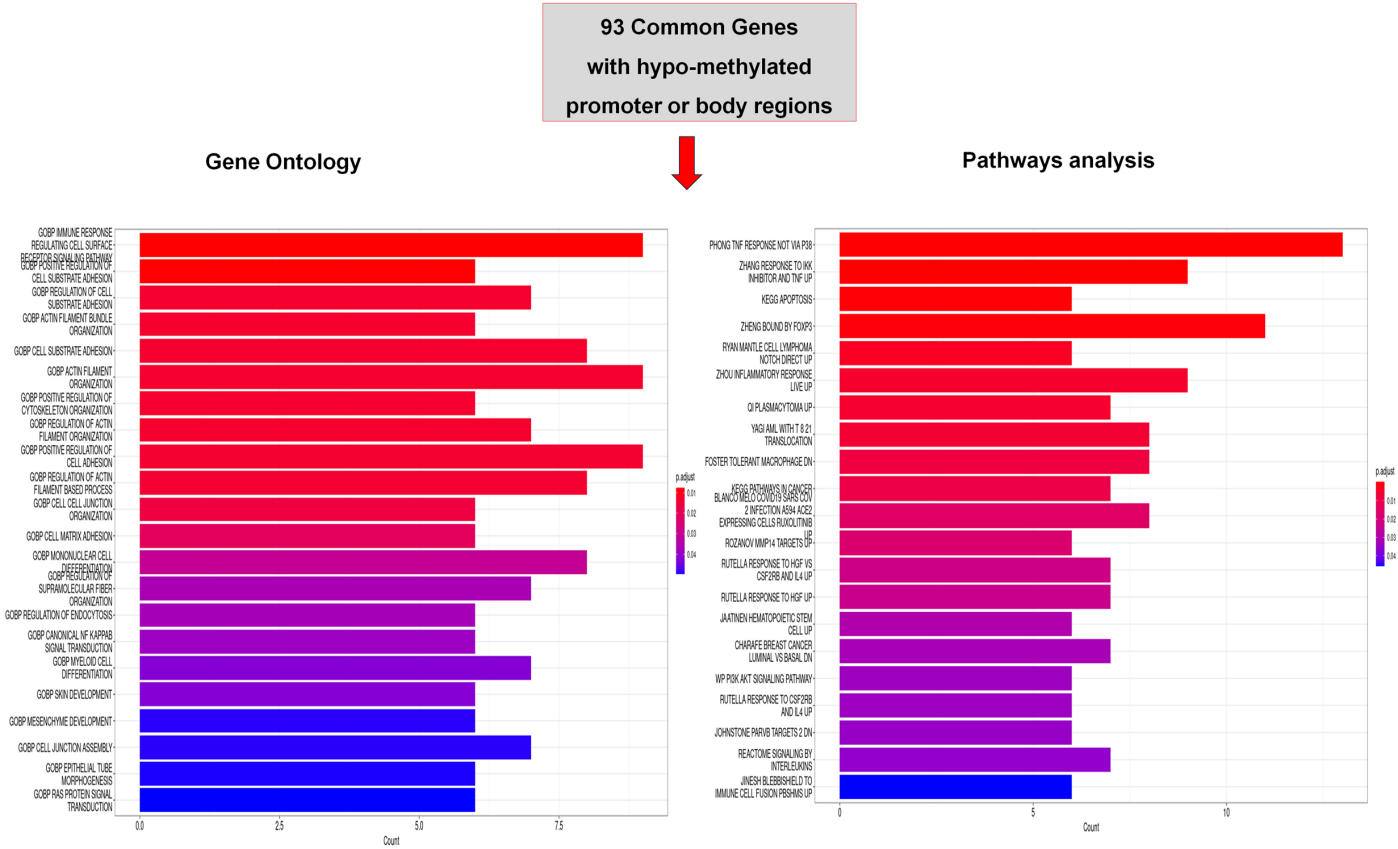
Functional analysis of common hypo-methylated dmGs. Gene ontology (GO) and Pathway analysis of common hypo-methylated dmGs of promoter and body regions belonging to 2 or 3 group intersections. The y-axis shows GO and pathway analysis and the x-axis represents the number of enriched genes. The color scale shows the significance of each GO or pathway showing the − log10 (adjusted *p*-value) with smaller *p*-value (red) representing more significant enrichment.

To understand which biological functions were associated with methylation changes in HSPCs treated with AML derived-EVs, we performed a functional analysis of 93 hypo-dmGs resulting from the intersection of two and three groups (**Figure 3**). Gene Ontology (GO) analysis indicated that these genes are involved in hematopoietic and immunologic process like IMMUNE_RESPONSE_REGULATING_CELL_SURFACE_ RECEPTOR_SIGNALING_PATHWAY (i.e. NFKB, TNIP and CD47), MONONUCLEAR_CELL_DIFFERENTIATION, MYELOID_CELL_ DIFFERENTIATION (i.e. SEMA4A, WDR1), or cell movement like POSITIVE_REGULATION_OF_CELL_ADHESION (i.e. CD47, SMAD3, MTSS1, WDR1), (**Supplementary Table 5**). Moreover, pathway analysis identified different inflammation cell signaling processes like PHONG_TNF_RESPONSE_NOT_VIA_P38, ZHANG_RESPONSE_TO_IKK_INHIBITOR_AND_TNF_UP and ZHOU_INFLAMMATORY_RESPONSE_LIVE_UP (i.e. TNFRSF10B, SMAD3, TNIP2, DSE, B4GALT5 and NFKB) and cancer processes like KEGG_PATHWAYS_IN_CANCER including AML and lymphoma (i.e. SMAD3, JAK1, PIK3R5, NFKB1, SLA) (**Supplementary Table 6**).

For the hyper-dmGs common to two and three groups, it was not possible to perform a functional analysis due to the low number of genes.

### 3.3 AML-EV induced dmGs were translated in differential expressed genes specifically involved in NF-kB pathway, in Interleukin mediated-signaling and toll like receptor cascade

**Figure 4:**
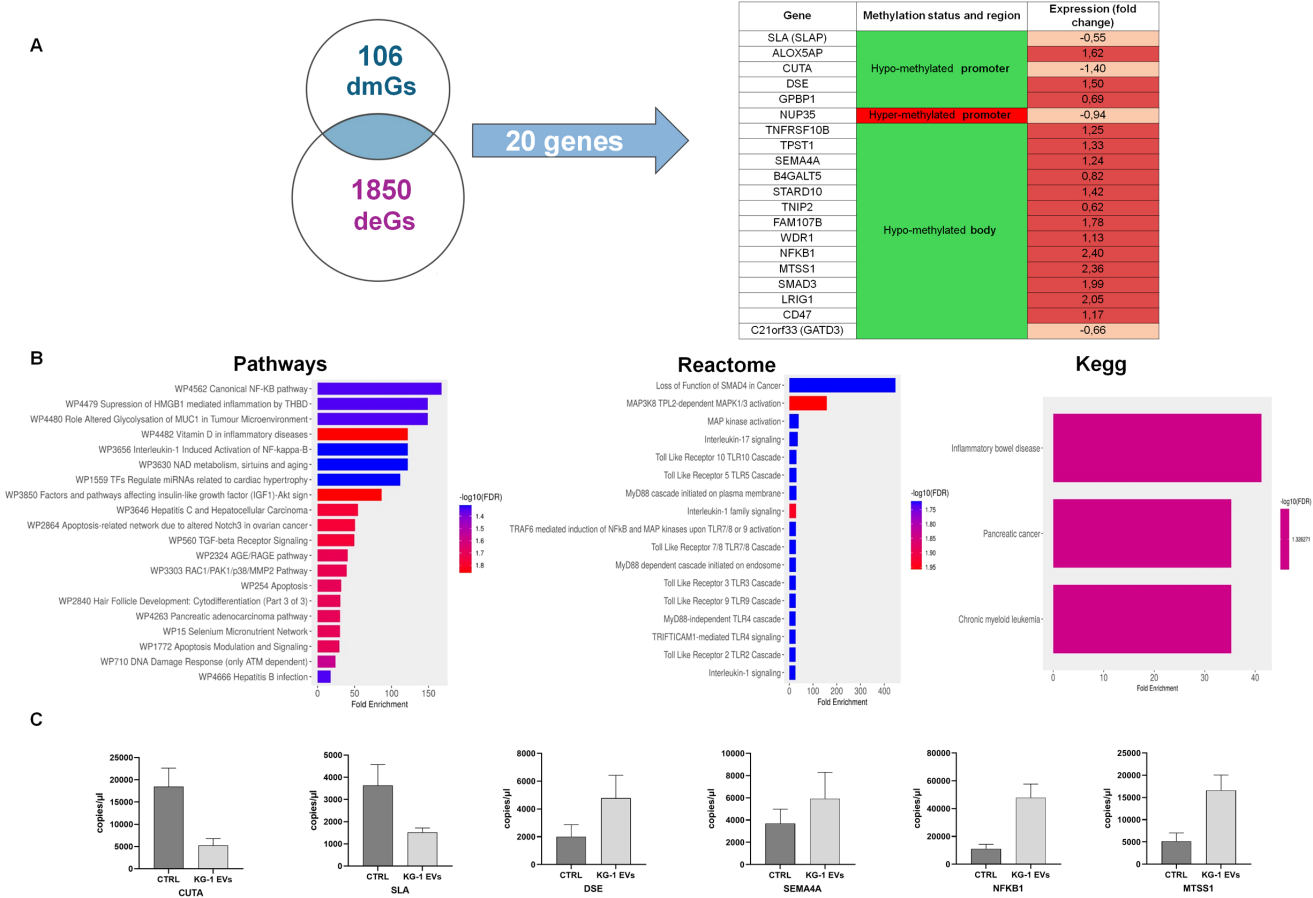
Identification and functional analysis of common genes between different methylated Genes (dmGs) and different expressed genes (deGs). **(A)** Venn diagram shows the number of common genes (n = 20) between 106 dmGs and 1850 deGs. The table shows the 20 common genes, their methylation and gene expression status. **(B)** Pathways, Reactome and Kegg analysis of 20 common genes. The y-axis shows Pathways, Reactome and Kegg analysis and the x-axis represents the number of enriched genes. The color scale shows the significance of each GO or pathway showing the − log10 (adjusted *p*-value). **(C)** Absolute quantification (copies/μl) by ddPCR of CUTA, SLA, DSE, SEMA4A, NFKB1 and MTSS1 mRNAs in HSPCs treated (KG-1-EVs) and not (Ctrl) with AML EVs. Values represent results from three independent experiments.

Transcriptional profiling represents the proximal readout of epigenetic control mechanisms, including DNA methylation. We adopted this approach in our study by crossing dmGs from hypo- and hyper-methylated promoter and body regions with differentially expressed genes (deGs) identified by previous gene expression profiling (GEP) of the same AML derived EV-treated versus untreated HSPCs (21) (**Figure 4**). The intersection between 106 dmGs and 1850 deGs (adjusted *p-*value< 0.05) allowed to identify 20 common genes to both analysis (**Figure 4A**). Among these, 5 genes had hypo-methylated promoter and of those 2 were down- and 3 were up-regulated in EV-treated versus untreated HSPCs; 14 genes had hypo-methylated body and of those only one is down-regulated while all the others are up-regulated in the treated HSPCs compared to the control; the last gene had hyper-methylated promoter and was down-regulated in the GEP (**Figure 4A**).

Enrichment analysis on these 20 genes showed their involvement in canonical NF-kB pathway, in inflammation, in IL-1 and -7 mediated-signaling and Toll like receptor cascade (TLR2, 3, 4, 9) and, of note, in cancer (**Figure 4B**).

To confirm their expression, ddPCRs were performed on six mRNAs, confirming that CUTA and SLA were down-regulated, while DSE, SEMA4A, NFKB1 and MTSS1 were up-regulated in EV-treated HSPCs (**Figure 4C**).

## 4 Discussion

**Figure 5:**
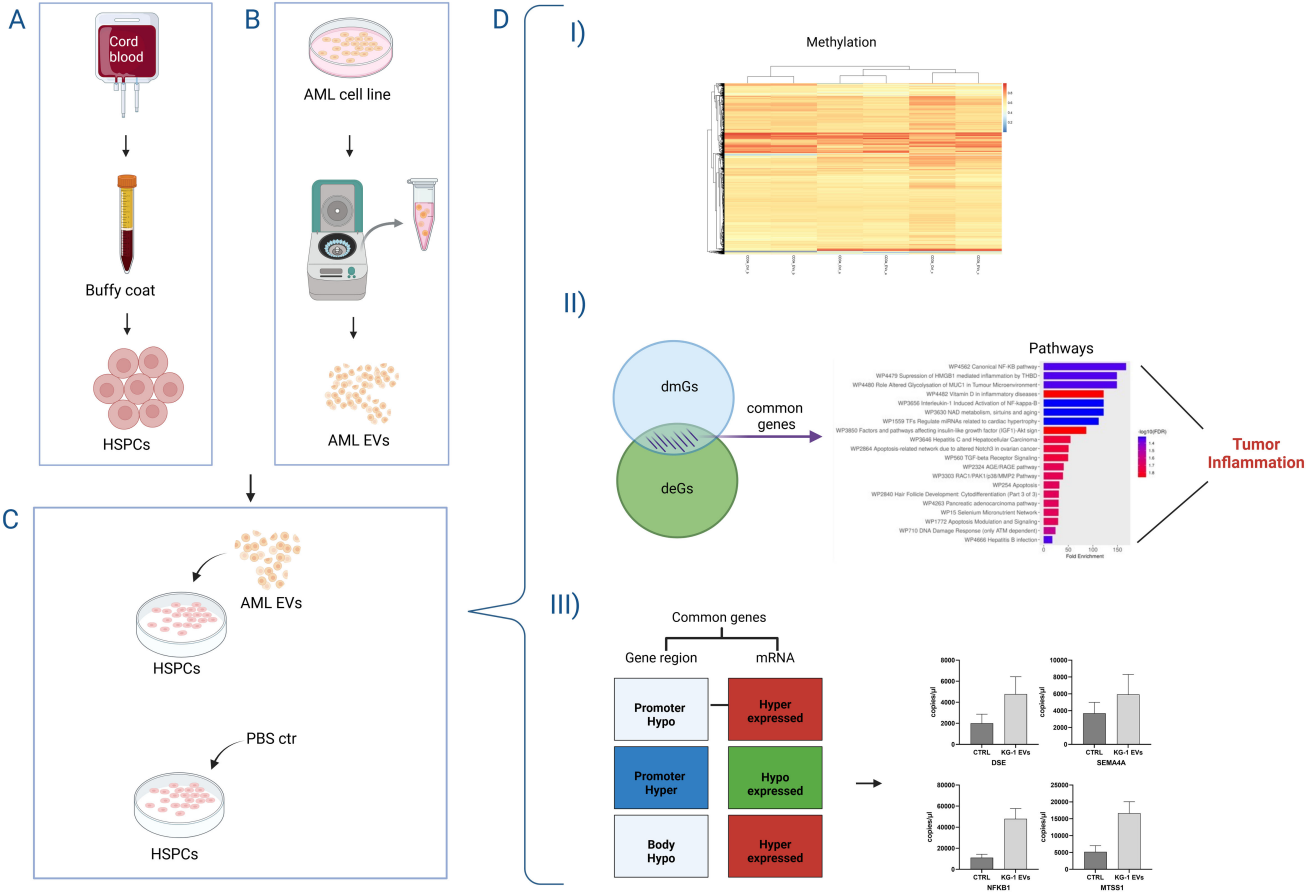
Representative cartoon of the effects of AML derived-EVs on DNA methylation of healthy primary Hematopoietic Stem and Progenitor Cells (HSPCs). HSPCS were obtained from cord blood bags by buffy coat **(A)** and AML-derived EVs were purified from AML cell line surnatant **(B)**. After the HSPC-exposure to AML-EVs or PBS ctr **(C)**, HSPCs showed **(D)** I) modification of DNA methylation, II) both differentially methylated and expressed genes (dmGs and deGs) involved in tumor inflammation and III) a gene expression directly affected by DNA methylation modifications.

Abnormal DNA methylation is often found in malignancies of the blood system, including AML, which exhibits a genome-wide hypo-methylation and an abnormal hyper-methylation of CpG islands. However, full understanding of the role of DNA methylation alterations in the evolution of AML is incomplete because the precise mutational consequences of hypo- or hyper-methylation have not so far been systematically evaluated (26, 27). In this perspective, our work aims to understand if leukemic EVs can somehow interfere in HSPCs epigenetics modifying their DNA methylation. In particular, we used an *in vitro* AML “pure” model to investigate, for the first time, the effects of AML derived-EVs on DNA methylation of healthy primary HSPCs demonstrating that this exposure induced *i*) the modification of DNA methylation, *ii*) the identification of dmGs involved in immunological processes like the inflammation and in cancer, *iii*) an DNA methylation mediated-effect on gene expression (**Figure 5**).

The first result observed after 20 hours of treatment with leukemic EVs, is that treated HSPCs (group a, b and c) maintained the original identity of the respective ctrl, clustering close to their parental cells. It means that all samples clustered by subject and not by treatment and this is an expected data that could be explained by the nature of primary cells. A similar trend was reported in our previous article, where the methylation of allogeneic HSPCs from different donors clustered close together to their respective time points after transplantation (22). This distribution had ensured that the subsequent analysis was conducted keeping each group separate from others. Moreover, differential analysis between EV-treated and untreated HSPCs of each individual group showed differences in the methylation signature (dmCpGs/dmGs) with a prevalence of hypo-methylation, particularly, in the gene body regions. Of note, cancer cells exhibit a global hypo-methylated genome which collectively leads to genomic instability and aberrant gene expression (28).

Our findings add to the few studies that highlighted EVs influence DNA methylation (16).

To date, most studies on epigenetic mechanism mediated gene regulation have focused on methylation changes in the promoter region. In particular, genome-wide methylation studies have shown that DNA methylation in the gene body is largely involved in the regulation of the expression of many genes and is closely related to the appearance and progression of malignant tumors (28). For this reason, in this work we studied the differences of methylation in both the promoter and gene body regions, identifying a prevalent hypo-methylation genes for both regions in all groups. DNA hypo-methylation in HSPCs indicates their differentiation into various hematopoietic progenitors. Specifically, demethylation has been demonstrated in the differentiation transition from hematopoietic stem cells to common myeloid progenitor cells and, finally, to the terminal committed erythroblast (29, 30).

In our study, HSPCs were treated with EVs derived from leukemic cells, therefore DNA hypo- or hyper-methylation of treated HSPCs respect to untreated ones could be due to the activation or deactivation of carcinogenic mechanisms. Alterations in DNA methylation have in fact been found in various types of cancer. In general, cancer cells present a global hypo-methylated genome with specific CpG island hyper-methylation which collectively leads to genomic instability and aberrant gene expression (26, 31).

Furthermore, as previously indicated, the regulation of DNA methylation guides normal hematopoiesis, but its alteration can often lead to the development of blood tumors. Literature data indicated that aberrant DNA methylation patterns during hematopoiesis linked to the dysfunction of DNA methylation-related enzymes promoting the transformation of HSPCs into leukemic cells (32).

Analyzing the hypo-methylated genes common to three groups we identified a series of functions. The main functions are cellular differentiation, including myeloid differentiation, functions related to cell adhesion or movement, inflammation and finally different cancer related pathways, including AML. All these functions could be related to each other, in fact it is known that inflammation influences the activation of hematopoiesis, but chronic inflammation, with the continuous activation of inflammatory cytokines, instead leads to damage to HSPCs, resulting in BM failure or the development of leukemia such as AML (33). Interestingly, it is known that the activation and the deactivation of inflammatory signaling pathways could be also regulated by DNA methylation. Many association studies across the epigenome, global DNA methylation patterns and candidate gene analysis suggest that inflammation is directly linked to global hypo-methylation of the genome (34). In this context, no information is present about a link between DNA methylation/inflammation and EVs in AML.

At this point we could hypothesize that AML-EVs directly altered the DNA methylation of HSPCs, modifying the expression of genes involved in inflammatory processes, differentiation and cell movement, probably in a neoplastic sense. We and other researchers have already demonstrated that AML-EVs are able to induce inflammation in the BM microenvironment by promoting the suppression of normal hematopoiesis, both *in vivo* and *in vitro*, transferring inflammatory related microRNA including miR-155 and miR-181a, but here it is highlighted that AML-EVs induce inflammation also by modifying DNA methylation (19, 21, 35).

The crossing between methylation and gene expression data carried out on the same samples allowed us to confirm this hypothesis, in fact genes common to the two analyses are involved in many of the previously mentioned functions. Specifically, enrichment analysis restricted their involvement in canonical NF-kB pathway, inflammation, interleukin and Toll like receptor (TLR) mediated-signaling and, of note, in cancer.

The transcription factor NF-kB, for example, regulates inflammatory responses but also plays pivotal roles in immune homeostasis and chronic inflammation, especially autoimmune diseases, tumorigenesis, chronic inflammatory diseases and aging (36, 37). Sema4A is a semaphorin expressed in immune cells and plays critical roles in many processes including cell-cell interactions, immune cell activation, differentiation and migration (38). Moreover, Sema4A and NF-kB appear to be linked. In particular, increase of NF-kB activated an increase of Sema4A which in turn up-regulated NF-kB, facilitating the development of lung cancer and osteoarthritis (39). In addition, all TLR signaling pathways (found in our enrichment analysis), culminate in activation of the transcription factor NF-kB (40). SLA (Src-like-adaptor) is an inhibitor of T and B cell receptor signaling. Furthermore, SLAP is associated with several oncogenic signaling pathways and its knock-out affects both B- and T-lymphocyte development in children with acute lymphoblastic leukemia (41, 42).

We next assessed the potential relationship between methylation status and gene expression in EV treated HSPCs reporting positive and negative correlation among two analyses. Surprisingly, the 20 genes identified from the intersection of two analyses are almost all over-expressed and hypo-methylated either in gene body or in promoter regions. Only two genes, SLA and CUTA, were down-regulated with hypo-methylated promoter. Generally, it was accepted that promoter hypo-methylation induces the activation of transcription, instead gene body methylation suppresses gene expression. It has been found that demethylation of the genome results in the appearance of many new active enhancers, evenly distributed across gene bodies and mediates the up regulation of gene expression, suggesting that gene body methylation silences enhancer function (43). This is mainly associated with reduced chromatin accessibility and reduced binding of transcription factors (28, 44). Therefore, in line with these findings, our results showed that genes with hypo-methylated promoter (e.g., ALOX5AP DSE) and body were actively transcribed. Overall, gene body methylation patterns and their biological effects are still key questions that need to be solved.

All these data allow us to hypothesize that leukemic cells can modify the epigenetic structure of healthy HSPCs through EVs. Our study, like others, reviews the vision of HSPCs as passive spectators physically displaced by the niche occupation of leukemic cells, demonstrating that healthy stem cells change their epigenome in contact with the leukemic EVs and acquiring an inflammatory state.

In conclusion, this study is the first proof-of-concept where it was demonstrated that AML-EVs directly modify the epigenetic structure of HSPCs by inducing an inflammatory state.

## Data Availability

The datasets presented in this study can be found in online repositories. The names of the repository/repositories and accession number(s) can be found below: GSE269215 (GEO).

## Supplementary material

Supplementary Figure 1 HSPC methylome analysis after AML-EVs treatment in group a. **(A)** Distribution of hypo- and hyper-methylated CpGs in DNA sites including OpenSea, Shelf (N and S), Shore (N and S) and Island. **(B)** Distribution in percentage and in number of hyper- and hypo-methylated CpGs located at different promoter and body regions. TSS200, TSS1500 and 5’UTR identified promoter, while 1^st^ exon, gene body, 3’UTR and exon boundaries were included in body region.

Supplementary Figure 2 HSPC methylome analysis after AML-EVs treatment in group b. **(A)** Distribution of hypo and hyper methylated CpGs in DNA sites including OpenSea, Shelf (N and S), Shore (N and S) and Island. **(B)** Distribution in percentage and in number of hyper- and hypo-methylated CpGs located at different genomic regions. TSS200, TSS1500 and 5’UTR identified promoter, while 1^st^ exon, gene body, 3’UTR and exon boundaries were included in body region.

Supplementary Figure 3 HSPC methylome analysis after AML-EVs treatment group c. **(A)** Distribution in percentage and in number of hypo and hyper methylated CpGs in DNA sites including OpenSea, Shelf (N and S), Shore (N and S) and Island. **(B)** Distribution of hyper- and hypo-methylated CpGs located at different genomic regions. TSS200, TSS1500 and 5’UTR identified promoter, while 1^st^ exon, gene body, 3’UTR and exon boundaries were included in body region.
