# Supplementary material for: Acute myeloid leukemia-derived extracellular vesicles induced DNA methylation changes responsible for inflammatory program in normal hematopoietic stem progenitor cells
Source: Front Immunol. 2025 Apr 10;16:1569159. doi: 10.3389/fimmu.2025.1569159 (PMC12018244; doi:10.3389/fimmu.2025.1569159)
Supplement: Supplementary Figure 1 — HSPC methylome analysis after AML-EVs treatment in group a. (A) Distribution of hypo- and hyper-methylated CpGs in DNA sites including OpenSea, Shelf (N and S), Shore (N and S) and Island. (B) Distribution in percentage and in number of hyper- and hypo-methylated CpGs located at different promoter and body regions. TSS200, TSS1500 and 5’UTR identified promoter, while 1st exon, gene body, 3’UTR and exon boundaries were included in body region. [file Presentation1.pptx]

## Slide 1
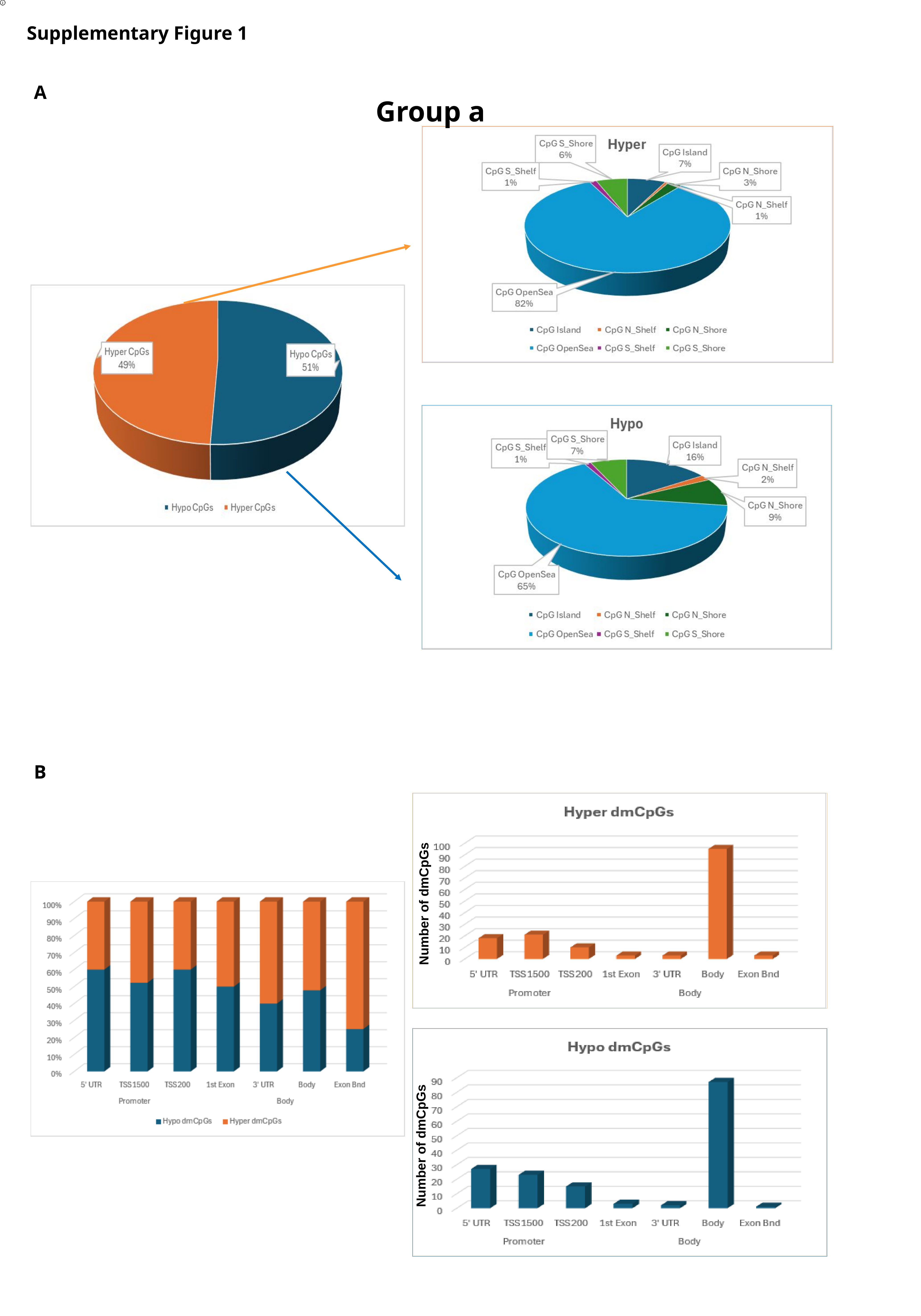

Supplementary Figure 1
A
Group a
B
Number of dmCpGs
Number of dmCpGs

## Slide 2
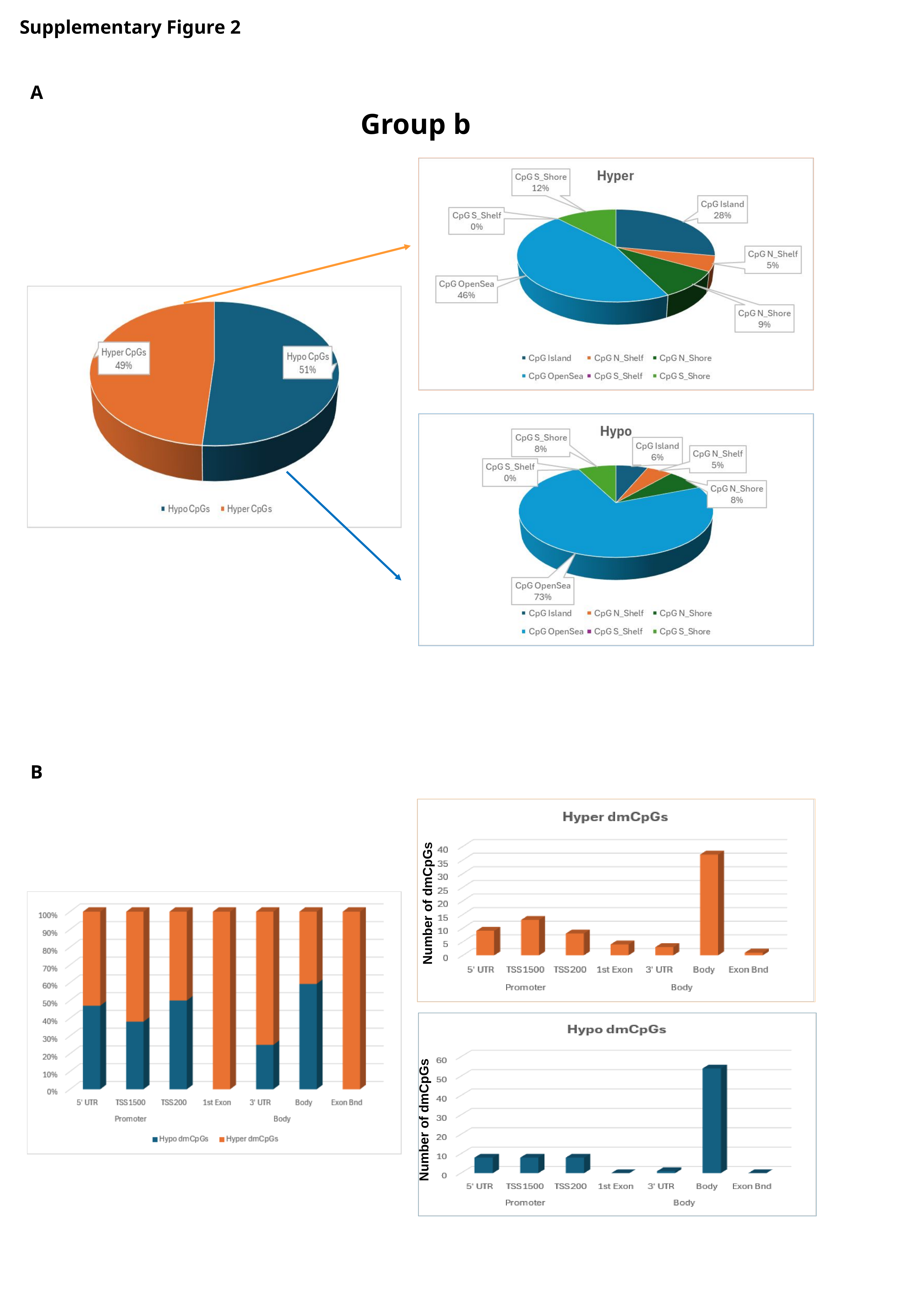

Supplementary Figure 2
A
Group b
B
Number of dmCpGs
Number of dmCpGs

## Slide 3
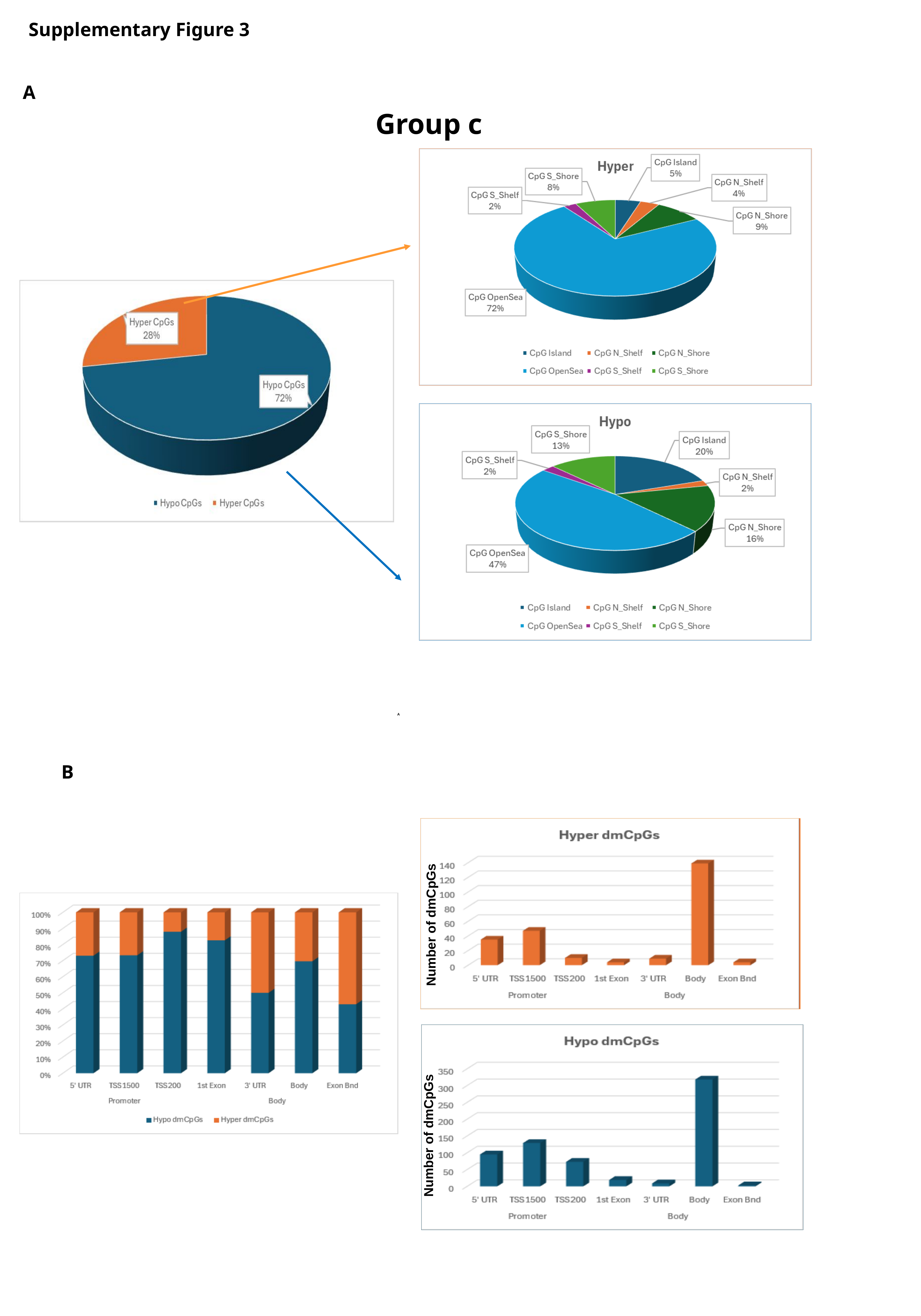

Supplementary Figure 3
A
Group c
A
B
Number of dmCpGs
Number of dmCpGs
